# E-education in pathology including certification of e-institutions

**DOI:** 10.1186/1746-1596-6-S1-S11

**Published:** 2011-03-30

**Authors:** Klaus Kayser, Robert Ogilvie, Stephan Borkenfeld, Gian Kayser

**Affiliations:** 1 UICC-TPCC, Charite, Charite Platz 1, 10118 Berlin, Germany; 2 Medical University of South Carolina, Charleston, SC 29403, USA; 3 IAT, Langgewann 39, D-69121 Heidelberg, Germany; 4 Institute of Pathology, University Freiburg, Breisacher Str. 15a, D-79106 Freiburg, Germany

## Abstract

E–education or electronically transferred continuous education in pathology is one major application of virtual microscopy. The basic conditions and properties of acoustic and visual information transfer, of teaching and learning processes, as well as of knowledge and competence, influence its implementation to a high degree. Educational programs and structures can be judged by access to the basic conditions, by description of the teaching resources, methods, and its program, as well as by identification of competences, and development of an appropriate evaluation system. Classic teaching and learning methods present a constant, usually non-reversible information flow. They are subject to personal circumstances of both teacher and student. The methods of information presentation need to be distinguished between static and dynamic, between acoustic and visual ones. Electronic tools in education include local manually assisted tools (language assistants, computer-assisted design, etc.), local passive tools (slides, movies, sounds, music), open access tools (internet), and specific tools such as Webinars. From the medical point of view information content can be divided into constant (gross and microscopic anatomy) and variable (disease related) items. Most open access available medical courses teach constant information such as anatomy or physiology. Mandatory teaching resources are image archives with user–controlled navigation and labelling, student–oriented user manuals, discussion forums, and expert consultation. A classic undergraduate electronic educational system is WebMic which presents with histology lectures. An example designed for postgraduate teaching is the digital lung pathology system. It includes a description of diagnostic and therapeutic features of 60 rare and common lung diseases, partly in multimedia presentation. Combining multimedia features with the organization structures of a virtual pathology institution will result in a virtual pathology education institution (VPEI), which can develop to a partly automated distant learning faculty in medicine.

## Introduction

Different compartments of medical education can be distinguished either in relationship to the already collected competence of the students (undergraduate, postgraduate, specialist), or to the subject of teaching (anatomy, physiology, disease – oriented), to the presentation of the information (passive, interactive, closed, open), to the presentation of the disease (bedside teaching, basic symptoms), to practical performance (dummies, action under supervision), or to online interaction (expert consultation, virtual slide (remote control) performance). The general properties of a teaching–learning process have been described by several authors; however, specific conditions in performing an optimized arrangement are not in agreement [1-4]. The accreditation of teaching institutes such as universities or medical schools in Europe has been standardized by the Bologna process [5], which regulates the standards of teaching and education at the high school and university level [5]. An international comparison of the students’ competences in relation to the taught courses should be obtained by the introduction of credits, i.e., scores that a student receives once he has successfully passed certain classes. The formal conditions have been fixed and internationally agreed to; however, the actual implementation, i.e., the actual quality of teaching and learning still remains uncertain. Independently from the Bologna process several universities introduced teaching and learning modules that take advantage of the internet, i.e., its open and standardized access [6-8].

Which conditions e-education systems have to be met in order to provide standardized and acknowledged services that are in agreement with the Bologna process? What are their specific conditions, and how can they be derived from conventional systems designed for both medical undergraduate and postgraduate education?

Basic conditions and principles of e- educational program:

Education is,in general,a transfer of information from a teacher or institution to students or humans who want to receive and practice this information [2,9-11].

Adequate teaching and learning can be analyzed in terms of

1) performance (methods and program)

2) resources

3) outcome of learning (knowledge and competence)

4) quality evaluation and assurance (accreditation)

**Figure 1:**
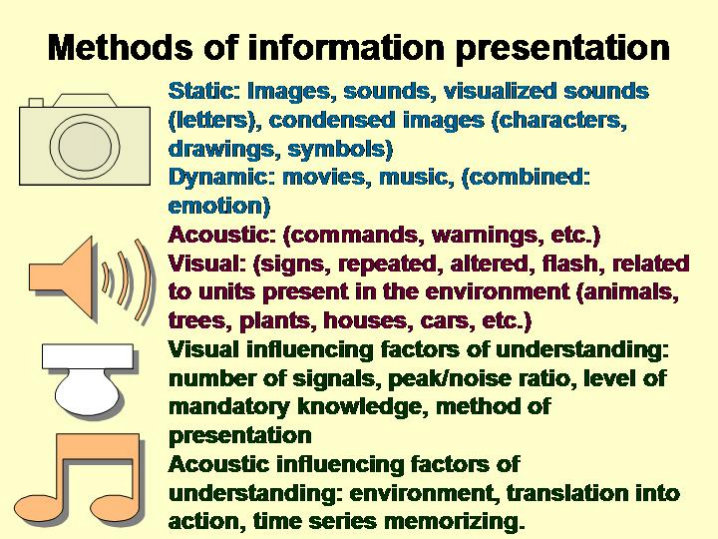
Differences between acoustic and visual information. Both are often biased if transferred from one into the other.

Adequate performance or information transfer requires that the level of submitted information is adjusted to the pre-existing knowledge of the students, otherwise the students will not benefit from the education either by not understanding the issue, or not adding new information to their knowledge [11]. Adequate performance is usually assured by sequential arrangement of classes, lectures, etc. with corresponding tests of students at the beginning and end of the classes. Classic education usually includes both, acoustic and visual information transfer. Acoustic information transfer is the backbone of classic education, which is summarized in the well-known sentence of “hearing his teacher’s voice” [11]. Acoustics allow a dynamic information transfer that is difficult to perform with movies or images (if viewed without acoustics). The differences of acoustic and visual communication are discussed in detail in [11,12]. These difference play an important role in e-education, as it is commonly based primarily on visual, and less on acoustic communication (see figure 1).

Aspects of human perception which are of importance in understanding the teaching– associated increase of competence are also related to the presentation of information, because perception is time or speed associated. It allows the student to eventually reach a level of competence which is higher compared to that of his teacher [11]. E-education should keep in mind that learning by still images remarkably differs from that of learning by speech or movies [11,13-15]. Electronic presentation of information for teaching purposes can be of passive (television, radio station, internet, etc.), of primarily passive (multiple choice tests, internet forums, electronic shopping, etc.) or of mainly active (games, chats, programming) nature. Independently from its formal nature, electronic presentation of information can be quantitatively measured at its source. At the side of its receiver it can, in addition, be classified into different distinct levels, which might be of more complex and higher stage compared to those at the side of the sender. In this case, the acquired knowledge possesses higher information content than presented, and is a quality measure for the teaching procedure.

Resources of e–education include image archives (microscopy, radiology, ultra sound, etc.), collections of drawings and schemes (anatomy, physiology), movies (animal experiments, patient’s behaviour, etc.), animations (simulation of functions in physiology, tissue growth, interactions of macromolecules, etc.), remote control performance (robotic microscopy, robotic surgery, etc.), and electronic simulation. They are subject related, and can be divided into constant information (such as the knowledge of anatomy, physiology, cellular interactions, etc.), and into those that can change by time (recognition and classification of diseases, diagnostic procedures, therapeutic regimes, etc.).

Outcome of learning is graded by tests that should assure that the student possesses the knowledge and competence either to enter the next class, or to practice the acquired information. In medicine both knowledge of diagnostics and therapy, and competence which is the correct and secure application of the taught methods are important. The quality of the applied tests can be judged by formal and content related parameters. The quality evaluation should focus on potential bias (exclusion of preference of certain students), individuality of the performed tests (not known to the students), and the level of requested knowledge (credit related).

Final accreditation of the university or medical school depends again on formal and content related items, which can be formal described by a tuning process [5]. In Europe, the analysis of the resources, performance, and obtained students’ knowledge and competence is handled by specific accreditation institutions which are commercially oriented [5]. We leave it to the opinion of the reader whether it is scientifically justified to transfer duties of significant social and human impact to commercially oriented institutions. Obviously, the arrangement is compatible to the trend of our society to strengthen the impact of an individual on the costs of the society.

## Medical requirements of e-education in tissue – based diagnosis

Description and analysis of disease related morphology is probably one goal in e–education. According to expense and the benefit or potential use and distribution, systems that focus on normal histology (anatomy) or physiology are easier to implement than those that are designed to teach diseases. Histology and anatomy did not change during the last one hundred years, and probably will not for the next century. Thus, images and text have to be prepared only once, and can be used for all correspondent undergraduate classes in the future. The preparation of disease – related lectures is time dependent as classification and knowledge change fast [8,14,16,17]. Such a system requires nearly daily maintenance and direct contact with the latest research results [11,18,19]. Not all “latest research results” can be confirmed by additional studies. Therefore, experience and intuition are necessary to detect the real important results.

## Implemented systems

**Figure 2:**
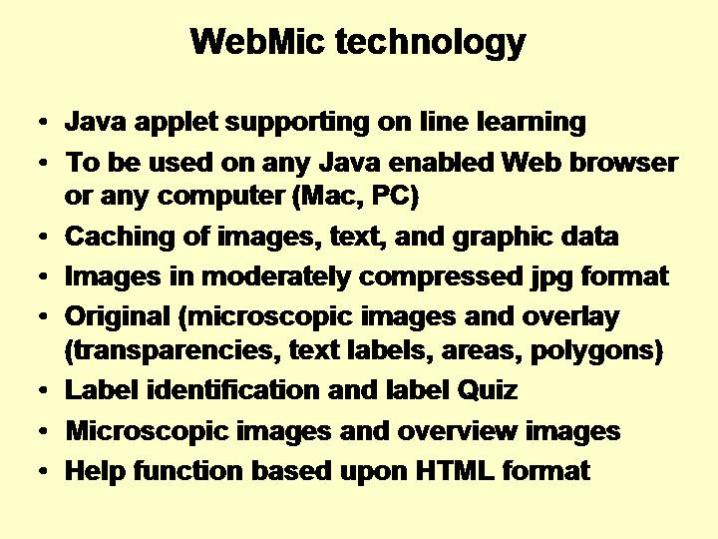
WebMic technology is based upon Java applets.

**Figure 3:**
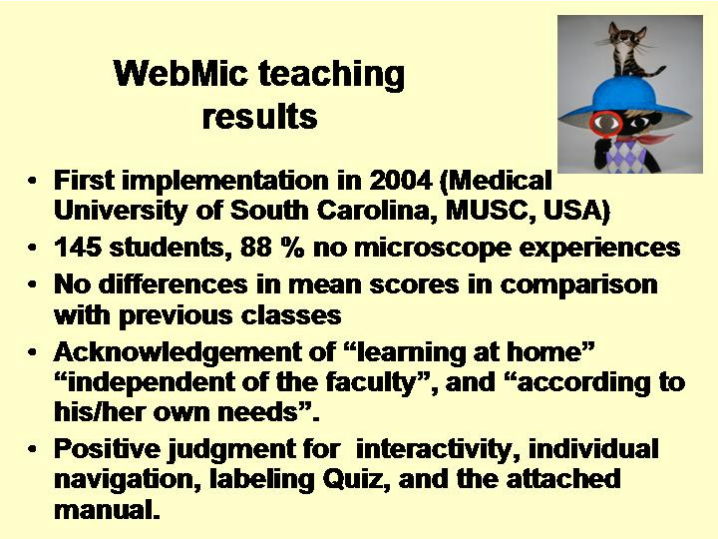
Obtained results when teaching with the WebMic

In microscopic anatomy, an example of one fully mature system for e–education is WebMic, a web-based histology learning program along with the ‘WebMic Study Guide: Learning Histology Step by Step’ [20,21]. WebMic has been designed for teaching practical histology to undergraduate students at universities and graduate students in medical schools. It consists of still (jpeg) images with annotations, a related text, appropriate tests, and a manual [21]. Its technology is listed in figure 2. Java applets to be used on any Java enabled Web browser support on-line learning. Images, text, and graphic data are held in cache. In addition to labels and quizzes interactive measurements (lines, areas, polygons) can be performed. The system was implemented at the Medical University of South Carolina, and tested on 154 students (figure 3) who received the same mean scores as their colleagues of previous classes, i.e., who are trained on conventional microscopes and in the conventional manner [20]. The system has been recently extended to an acoustic teaching course that includes dynamic lectures in histology created with Adobe Presenter along with virtual slides [22].

**Figure 4:**
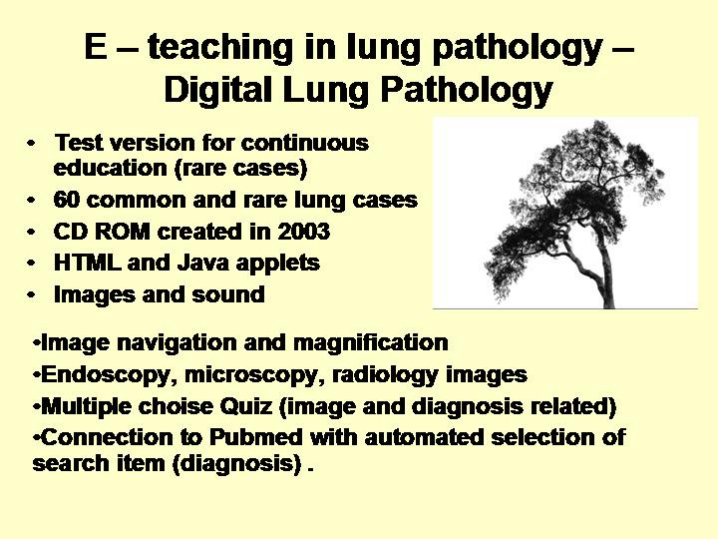
Features of the e – teaching and reference system /digital lung pathology/

At present complete e-education systems that teach pathology are not available to our knowledge (despite electronic versions of text books). However, a pilot study on digital lung pathology has been published a few years ago [23]. It has been designed as test version for continuous education in lung pathology and includes a total of 60 rare and common lung diseases. Its features are shown in figure 4. It possesses two different quizzes (image and diagnosis based multiple choice), and a user friendly internet search access to the medical library of the National Institute of Health (NIH) [24].

## Medical virtual education institutes and perspectives

Until today, a virtual pathology education institution (VPEI) does not exist to our knowledge. The design of such an institution should include undergraduate education, especially in histology and anatomy, physiology, and post graduate education which will be probably combined with continuous education in medical specialities. Postgraduate and continuous education should include training courses similar to the advanced WebMic and stratified (disease oriented, open) image databanks as well as expert consultation modules. Evaluation of image quality and diagnostic accuracy are prerequisites to further develop such a system to a fully automated virtual microscope. An international advisory board and continuous maintenance are further essentials in order to provide reliable credits and appropriate accreditation. The fast development of digital pathology will probably also promote the implementation of VPEIs in the near future.

## Competing interests

The authors declare that they have no competing interests.
